# Depletion of dietary aryl hydrocarbon receptor ligands alters microbiota composition and function

**DOI:** 10.1038/s41598-019-51194-w

**Published:** 2019-10-11

**Authors:** Kyle M. Brawner, Venkata A. Yeramilli, Lennard W. Duck, William Van Der Pol, Lesley E. Smythies, Casey D. Morrow, Charles O. Elson, Colin A. Martin

**Affiliations:** 1 Department of Surgery, University of Alabama at Birmingham, Birmingham, Alabama United States; 2 Department of Medicine, University of Alabama at Birmingham, Birmingham, Alabama United States; 3 Center for Clinical and Translational Science, University of Alabama at Birmingham, Birmingham, Alabama United States; 4 Department of Cell, Developmental, and Integrative Biology, University of Alabama at Birmingham, Birmingham, Alabama United States

**Keywords:** Mucosal immunology, Microbiome

## Abstract

The intestinal microbiota is critical for maintaining homeostasis. Dysbiosis, an imbalance in the microbial community, contributes to the susceptibility of several diseases. Many factors are known to influence gut microbial composition, including diet. We have previously shown that fecal immunoglobulin (Ig) A levels are decreased in mice fed a diet free of aryl hydrocarbon receptor (AhR) ligands. Here, we hypothesize this IgA decrease is secondary to diet-induced dysbiosis. We assigned mice to a conventional diet, an AhR ligand-free diet, or an AhR ligand-free diet supplemented with the dietary AhR ligand indole-3-carbinol (I3C). We observed a global alteration of fecal microbiota upon dietary AhR ligand deprivation. Compared to mice on the conventional diet, family *Erysipelotrichaceae* was enriched in the feces of mice on the AhR ligand-free diet but returned to normal levels upon dietary supplementation with I3C. *Faecalibaculum rodentium*, an *Erysipelotrichaceae* species, depleted its growth media of AhR ligands. Cultured fecal bacteria from mice on the AhR ligand-free diet, but not the other two diets, were able to alter IgA levels *in vitro*, as was *F*. *rodentium* alone. Our data point to the critical role of AhR dietary ligands in shaping the composition and proper functioning of gut microbiota.

## Introduction

The aryl hydrocarbon receptor (AhR) is a transcription factor that was originally studied for its role in mediating the metabolism of its xenobiotic ligands. As a sensor of environmental chemicals, AhR is heavily expressed at barrier sites such as the skin, lungs, and gut^1^. More recently, physiological AhR ligands have been identified, including gut microbiota-derived tryptophan metabolites^2–4^ and dietary compounds^5^. A growing body of work illustrates the vital role of AhR in maintaining gut health and homeostasis. AhR antagonism leads to more severe chemically-induced colitis, and expression of AhR is reduced in intestinal tissue of ulcerative colitis (UC) patients^6^. The serum level of indole-3-propionic acid (IPA), an AhR ligand generated from microbial tryptophan catabolism, is decreased in both UC patients and in experimental murine colitis. Importantly, oral administration of IPA to mice ameliorated disease^7^. Mice deficient in caspase recruitment domain 9 (Card9), an inflammatory bowel disease (IBD) susceptibility gene, harbor a dysbiotic gut microbiota that is unable to metabolize tryptophan into AhR ligands, leading to more severe chemically-induced colitis^8^. Dietary sources of AhR ligands that are not directly produced by the microbiota are also capable of modulating the gut environment. Perhaps one of the best studied dietary sources of AhR ligands is indole-3-carbinol (I3C), which upon ingestion is converted by acid hydrolysis in the stomach to high-affinity AhR ligands such as indolo[3,2-b]carbazole (ICZ)^9,10^ and 3,3′-diindolylmethane (DIM)^11^. Dietary supplementation with I3C protects mice from developing colorectal cancer and ameliorates tumorigenesis already in progress^12^. In addition, mice on a diet low in AhR ligands harbor a higher gut bacterial load compared with mice on a control diet, and dietary I3C is sufficient to lower the bacterial load to normal levels^13^.

We have shown previously that mice fed a diet deficient in AhR ligands have lower levels of fecal immunoglobulin (Ig) A compared with mice on a conventional chow diet^14^. IgA is the predominant antibody isotype at mucosal sites, and throughout the body, more IgA is produced than every other antibody isotype combined^15^. Although it has long been known that IgA protects against infection from clear gastrointestinal pathogens such as *Salmonella typhimurium*^16^, *Vibrio cholera*^17^ and *Shigella flexneri*^18^, IgA coating of commensal microbes in human^19^ and mouse^20^ fecal samples has been described. Mouse experiments have indicated that proper IgA coating of gut commensals requires functional B cell selection and affinity maturation^21^, suggesting IgA coating of commensal microbes is largely a specific response rather than a product of cross-reactivity. In patients with IBD, the percentage of fecal microbes bound by IgA increases^22^. Because dysbiosis is a hallmark of IBD^23^, it has been speculated that IgA particularly targets pathobionts^24^, members of the microbiota with the potential to directly stimulate a potent inflammatory response. Coating of gut microbiota with IgA does not seem to inhibit bacterial growth or cause clearance, but is considered vital for proper physical containment of the microbes and for immune homeostasis^25,26^. Therefore, a deficiency in gut IgA for any reason, even in the absence of overt pathogens, can result in deleterious consequences for gastrointestinal health^27^.

Previous studies have demonstrated that certain pathogenic bacterial species^28,29^ as well as whole fecal microbiota from mice^30^ have the ability to degrade IgA. Here, we hypothesized that an AhR ligand-free diet would alter the composition of the microbiota, and that this altered microbiota directly contributes to the low fecal IgA levels characteristic of mice on this diet. We also sought to identify potential commensal species capable of shaping luminal IgA levels.

## Methods

### Animal husbandry and special diets

C57BL/6 mice were purchased from Jackson Laboratories (Bar Harbor, ME) and bred in our animal facility to obtain the mice used in this study. Special diets were created and administered as previously described^14^. In brief, the AhR ligand-free diet AIN-76A and AIN-76A supplemented with indole-3-carbinol (1000 parts per million)^31^ were purchased from Envigo. Mice were placed on their respective diets immediately after weaning, at approximately 2 weeks of age, and fecal samples were collected 8 weeks later. Animals were bred and maintained under specific pathogen-free conditions in Thoren Isolator racks under positive pressure. All experiments involving animals were approved by the Institutional Animal Care and Use Committee at the University of Alabama at Birmingham.

### 16S rDNA sequencing and bioinformatics analysis

DNA was isolated from fecal pellets using the ZR Fecal DNA MiniPrep Kit (Zymo Research, Irvine, CA, USA). PCR, sequencing, and bioinformatics analysis were performed as we have described previously^32^.

A BLAST analysis (National Center for Biotechnology Information) was performed on all of the *Erysipelotrichaceae* 16S rDNA sequences that were among the top 100 most abundant operational taxonomic units (OTUs) from each group of mice in the study. Sequences were searched against the 16S ribosomal RNA database and optimized for a megablast algorithm. Results are valid as of June 7, 2018.

### Culturing of fecal microbes

Fresh fecal samples from four mice on a conventional diet were pooled, fecal samples from three mice on the AhR ligand-free diet were pooled, and fecal samples from three mice on the AhR ligand-free diet supplemented with I3C were pooled. These pooled samples were weighed and resuspended in sterile PBS to a concentration of 200 mg/mL. Chopped meat carbohydrate broth (BD Biosciences, San Jose, CA, USA) was inoculated with 250 µl of this suspension and cultured at 37 °C overnight in an anaerobic chamber. The following day, cultures were aliquoted and stored at −80 °C until being used in IgA transcytosis and luciferase assays described below.

### Bacterial culture and preparation of bead-beaten extracts

*Faecalibaculum rodentium* strain ALO17 was purchased from Deutsche Sammlung von Mikroorganismen und Zellkulturen (DSMZ) (Braunschweig, Germany). Frozen isolates of *F*. *rodentium* were swabbed on a blood agar plate and incubated at 37 °C in an anaerobic chamber (day 0). On day 5, a single colony on the plate was transferred into a vial of chopped meat broth with carbohydrates (BD). After an additional 2 days, 1 mL of bacteria-containing broth was transferred to 2 tubes each of chopped meat broth with carbohydrates and 2 tubes each of chopped meat broth with no carbohydrates (Anaerobe Systems, Morgan Hill, CA, USA). L-Tryptophan (Sigma, St. Louis, MO, USA) was dissolved in PBS to create a 70 mM solution. Hydrochloric acid was added dropwise until tryptophan was fully dissolved. After the solution was sterile filtered, an appropriate volume was added to 1 tube of bacteria in chopped meat with carbohydrates and 1 tube of bacteria in chopped meat with no carbohydrates to achieve a tryptophan concentration of 0.6 mM. Therefore, four culture conditions were created: 1) *F*. *rodentium* in chopped meat broth with carbohydrates 2) *F*. *rodentium* in chopped meat broth with carbohydrates and exogenous tryptophan 3) *F*. *rodentium* in chopped meat broth with no carbohydrates 4) *F*. *rodentium* in chopped meat broth with no carbohydrates but with exogenous tryptophan. After a final 24-hour incubation, the optical density of each culture at 600 nm (OD_600_) was measured (0.759, 0.937, 0.468, and 0.474, respectively). After this measurement, broth samples were transferred to a 15 mL tube, centrifuged for 20 minutes at 3000 RPM, and supernatants collected and frozen at −80 °C until needed. To prepare bead-beaten extracts, the pellets were re-suspended in 500ul of PBS and transferred to 2 mL microcentrifuge tubes fitted with a rubber seal. BioSpec 0.1 mm glass beads (Fisher Scientific) were added to the tubes and tubes were beaten in a Mini Bead-Beater (Biospec Products, Bartlesville, OK, USA) for 1 minute. After beating, tubes were centrifuged at 13,000 RPM for 2 minutes and the extracts were collected. The amount of total protein in the extracts was quantified by the DC Protein Assay (Bio-Rad, Hercules, CA, USA). Extracts were stored at −80 °C until needed.

*Lactobacillus reuteri* strain PTA-6475 was purchased from American Type Culture Collection (ATCC) (Manassas, VA, USA). Frozen isolates were plated on a MRS agar plate and incubated in an anaerobic chamber at 37 °C for 48 hours. After this incubation, a single colony on the plate was inoculated into MRS broth and allowed to incubate for an additional 24 hours. The next day, the bacteria-containing broth was diluted 1:10 into 2 vials of fresh MRS broth and 2 vials of fresh carbohydrate-free peptone-tryptone water (Sigma). A volume of the tryptophan solution described above was added to 1 vial of MRS broth and 1 vial of peptone-tryptone water to achieve a tryptophan concentration of 0.6 mM. After a final 24-hour incubation, the vials were centrifuged and supernatants collected and frozen at −80 °C until needed.

### AhR ligand luciferase reporter assay

HCT116 cells that have been engineered to express luciferase under the control of the dioxin response element were kindly provided by Dr. Greg Kennedy at the University of Alabama at Birmingham and were seeded in 96-well white wall plates (Corning, Corning, NY, USA) at a density of 10,000 cells per well. Cells were cultured in Dulbecco’s modified Eagle’s Medium (DMEM) supplemented with 10% fetal bovine serum (FBS), 100 U/mL of penicillin, 100 µg/mL of streptomycin, nonessential amino acids, 2 mM L-glutamine, and 100 µg/mL of hygromycin, all from Gibco except for hygromycin (Invitrogen, Carlsbad, CA, USA). The next day, cells were treated with bacteria-conditioned or non-conditioned broth at a dilution of 1:8 by volume or with 50 µg or 100 µg total protein of *F*. *rodentium* extract. As a positive control, some cells were treated with 100 nM of 6-formylindolo[3,2-b]carbazole (FICZ, purchased from Sigma), a known AhR ligand. Dimethyl sulfoxide (DMSO), the FICZ vehicle, was used as the negative control. After a 6 hour incubation at 37 °C, supernatants were discarded and cells were rinsed with PBS. Cells were subsequently lysed and treated with luciferase substrate using the Luciferase Assay System (Promega, Madison, WI, USA). Light intensity was measured with a SpectaMax L luminometer (Molecular Devices, San Jose, CA, USA) and data collected with Softmax version 5.4.5 software (Molecular Devices).

### Colonic spheroid and transwell monolayer culture

Primary colonic epithelial stem cells were isolated, grown, and maintained as spheroids as previously described^30,33^. Briefly, mouse colon was harvested, washed with PBS, minced, and digested in a 2 mg/mL solution of collagenase type 1 (Gibco) for 50 minutes at 37 °C. Minced tissue in this solution was then filtered through a 70 µm cell strainer and filtered cells were centrifuged at 930 RPM for 5 minutes. Pelleted cells were then resuspended in Matrigel (BD Biosciences), plated onto a 24-well plate, and cultured in 50% L-WRN (*L* cells expressing *W*nt3a, *R*-spondin3, and *N*oggin) conditioned medium. The L-WRN medium was supplemented with 10 µM of the Rho-associated protein kinase (ROCK) inhibitor Y27632 and 10 µM of the transforming growth factor (TGF)-β type I receptor inhibitor SB431542, both from R&D Systems (Minneapolis, MN, USA). L-WRN medium was replaced every 2 days, and spheroids were passaged every 3 days.

Epithelial cell monolayers from these spheroids were generated as previously described^34^. Briefly, 3-day-old spheroids were washed in a solution of 0.5 mM EDTA in PBS, dissociated into single cells by incubation in 0.05% trypsin/0.5 mM EDTA solution (Gibco) for 3.5 minutes at 37 °C, washed in DMEM/F12 media (Sigma) containing 10% FBS (Atlanta Biologicals, Flowery Branch, GA, USA), and filtered through a 40 µm cell strainer. Pelleted cells were then resuspended in 5% L-WRN conditioned medium supplemented with 10 µM Y27632 inhibitor and counted. 50% L-WRN medium was diluted to 5% using Advanced DMEM/F12 (Gibco) supplemented with 20% FBS (Atlanta Biologicals), 100 U/mL of penicillin, 100 µg/mL of streptomycin, and 2 mM L-glutamine. Transwell inserts (Corning) were coated with Matrigel diluted 1:30 in PBS. After incubation at 37 °C for 20–30 minutes, the Matrigel/PBS solution was removed and the cells immediately seeded in the apical Transwell compartment at a density of 100,000 cells per insert. This was considered day 0.

### Cell treatments and IgA transcytosis assay

Cells in Transwell inserts were given fresh 5% L-WRN medium supplemented with 1 µg/mL lipopolysaccharide (LPS) (Sigma), 10 µM DAPT γ-secretase inhibitor (Millipore, Burlington, MA, USA), and 10 µM Y27632 inhibitor on days 1 and 2 of the monolayer culture.

IgA transcytosis assays were performed on day 3, as previously described^30^. Briefly, monolayers were removed from their treatment conditions and switched to fresh 5% L-WRN medium with 10 µM Y27632 inhibitor. 5 µg/mL IgA (BD Pharmingen) was added to the lower compartment. In the upper compartment, treatments included *F*. *rodentium* bead-beaten extract, supernatant conditioned by *F*. *rodentium*, or fecal bacteria cultures (all diluted 1:10 by volume). The extracts and conditioned supernatants derived from *F*. *rodentium* that were used for these experiments were generated from *F*. *rodentium* that had been grown in chopped meat broth with carbohydrates and no exogenous tryptophan. After incubating for 6 hours at 37 °C, apical supernatants were removed for IgA ELISA, cells in the Transwell were washed, transepithelial electrical resistance (TER) was measured, and RNA was collected from the cells (described below).

### TER measurements

TER was measured from monolayers growing in Transwell inserts using an epithelial volt-ohm meter (World Precision Instruments, Sarasota, FL, USA). Two measurements were recorded. An initial measurement was taken on day 3 immediately before the addition of bacterial samples and IgA to the top and bottom compartments, respectively. Immediately after the 6 hour incubation allowed for the transcytosis assay, a second TER measurement was taken. The background TER measured from medium in a Transwell with no cells was subtracted from the resistance measured from a Transwell containing cells and medium. Each Transwell was measured in triplicate and the average value recorded.

### Enzyme-linked immunosorbent assay

IgA levels in apical supernatants and mouse fecal samples were measured by ELISA, as we have previously described^14^.

### Gene expression analysis

Immediately following collection of apical supernatants and the second TER measurement on day 3, cells in Transwell inserts were lysed in TRIzol Reagent (Invitrogen) and RNA was isolated according to the manufacturer’s instructions. Complementary DNA synthesis was performed using 200 ng of RNA and the High Capacity cDNA Reverse Transcription Kit (Applied Biosystems, Foster City, CA, USA). Quantitative polymerase chain reactions were performed with TaqMan Fast Advanced Master Mix (Applied Biosystems). Both reverse transcription and qPCR were performed in a QuantStudio 3 thermal cycler (ThermoFisher, Waltham, MA, USA). Expression levels for each sample were determined in duplicate and normalized to expression of glyceraldehyde 3-phosphate dehydrogenase (*Gapdh*) by the ΔΔC_T_ method. The primers used were: *Gapdh*, assay ID Mm99999915_g1; and *Pigr*, assay ID Mm00465049_m1, both from ThermoFisher.

### Metabolomics analysis

#### Metabolite extractions from media

100uL culture supernatant or non-conditioned chopped meat broth was added to 600 µL of acetonitrile to a Phree column (Phenomenex, Torrence CA) to remove intact protein and phospholipid content. The vacuum manifold extracted supernatant was then removed and dried under N_2_ gas. 100 µL of 0.1% Formic Acid was added to ddH_2_0 for mass spectrometry evaluations.

#### NanocHiPLC-tandem mass spectrometry

An aliquot (5 µL) of each digest was loaded onto a Nano cHiPLC 200 µm × 6 mm ChromXP C18-CL 3 µm 120 Å reverse-phase trap cartridge (Eksigent, Toronto, Canada) at 2 µL/min using an Eksigent autosampler. After washing the cartridge for 5 min with 0.1% formic acid in ddH_2_0, the bound peptides were flushed onto a Nano cHiPLC column 200 µm × 15 cm ChromXP C18-CL 3 µm 120 Å (Eksigent, Toronto, Canada) with a 20 min linear (2–98%) acetonitrile gradient in 0.1% formic acid at 1000 nl/min using an Eksigent 400 NanoLC System. (Dublin, CA). The column was washed with 98% acetonitrile-0.1% formic acid for 5 min and then re-equilibrated with 2% acetonitrile-0.1% formic acid for 5 min. The SCIEX 5600 Triple-Tof mass spectrometer (SCIEX, Toronto, Canada) was used to analyze the protein digest. The IonSpray voltage was 2300 V and the declustering potential was 80 V. Ionspray and curtain gases were set at 10 psi and 25 psi, respectively. The interface heater temperature was 120 °C. Eluted metabolites were subjected to a time-of-flight 250msec survey scan from 50–1000 m/z to determine the top twenty most intense ions for MSMS analysis. Product ion time-of-flight scans at 50 msec were carried out to obtain the tandem mass spectra of the selected parent ions over the range from *m/z* 50–1000 using a rolling collision energy parameter to determine the best fragmentation energies per compound mass.

#### Processing data to XCMS online and metaboanalyst software

LC-MS data were processed using XCMS-Online (https://xcmsonline.scripps.edu/) to identify and align peaks occurring across all samples. The data was then exported into individual Excel.csv files for each sample containing the *m/z*, peak area and retention time of each metabolite ion. The exported files were then organized and uploaded to MetaboAnalyst version 3.5 (http://www.metaboanalyst.ca/) for statistical evaluations.

### Statistical analysis

Unless otherwise indicated, statistical significance between two groups was determined by two-tailed unpaired Student’s *t* test. Comparison of more than two groups was performed with one-way ANOVA followed by Tukey’s multiple comparisons test. Calculations were performed using GraphPad Prism software version 7.03. A *P* value of <0.05 was considered statistically significant.

## Results

### Fecal microbiota composition is altered is the absence of dietary AhR ligands

**Figure 1:**
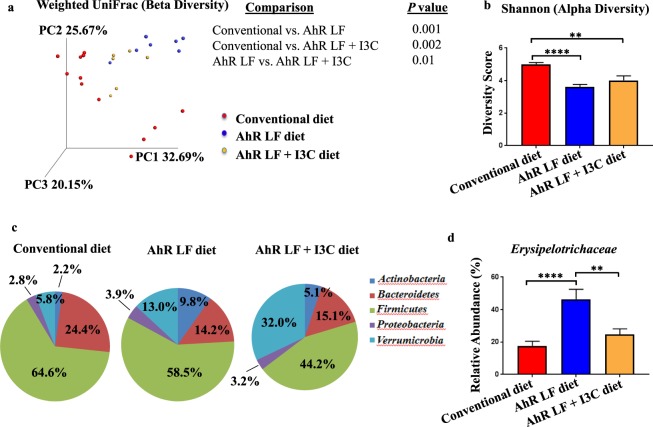
Fecal microbiota composition is altered in the absence of dietary AhR ligands. (**a**) Weighted UniFrac analysis of the global fecal microbiota composition from mice on a conventional diet (n = 12), mice on the AhR ligand-free (LF) diet (n = 7), and mice on the AhR LF diet supplemented with I3C (AhR LF + I3C diet) (n = 6). Each dot represents one mouse. Comparisons between any two given groups show the significance of the overall microbiota difference. Differences in overall microbiota composition among groups were calculated by a PERMANOVA test using Quantitative Insights into Microbial Ecology software. (**b**) Alpha diversity analysis of the fecal microbiota (Shannon index). (**c**) Average proportion of the major phyla present in fecal samples from mice on the indicated diet. (**d**) Relative abundance of *Erysipelotrichaceae*. Error bars represent mean ± SEM. Data for a-d are pooled from two independent experiments. **p < 0.01 ****p < 0.0001, one-way ANOVA with Tukey’s multiple comparisons test.

We have previously shown that mice fed an AhR ligand-free diet contain less fecal IgA than mice fed a conventional diet^14^. The reduction in fecal IgA in mice on the ligand-free diet led us to hypothesize that mice on this diet harbor a microbiota distinct from mice on a normal chow diet. Microbiota differences were confirmed by subjecting bacterial DNA in fecal samples to 16S rDNA gene sequencing. Weighted UniFrac distance analysis was employed. This distance measure takes into account the relative abundance of OTUs in a sample and accounts for evolutionary relatedness of OTUs in calculating the degree of similarity among samples. As seen in Fig. 1a, this analysis clearly shows a high degree of dissimilarity in the microbiota composition between mice on the conventional and AhR ligand-free diets. In addition, mice on the ligand-free diet on average possessed a less diverse microbiota compared to mice on a conventional diet, as revealed by the Shannon diversity index (Fig. 1b). The Shannon index is a measure of alpha diversity. A diversity score is calculated based on the number of OTUs (richness) in a sample and how evenly the relative abundance of the OTUs is distributed within a sample. The higher the richness and more even the distribution, the higher the alpha diversity score. Dietary supplementation of the known AhR dietary ligand I3C into the ligand-free diet altered global microbiota composition (Fig. 1a) but not intra-sample diversity (Fig. 1b) relative to mice on the AhR ligand-free diet.

We next sought to identify specific taxa that might be contributing to the global microbiota changes described above. We limited our search to taxa that met the following criteria: (1) The relative abundance is significantly altered in the AhR ligand-free diet group relative to the conventional diet group, (2) The relative abundance in the conventional diet group and AhR ligand-free + I3C diet group is similar, and (3) The relative abundance is at least 1% in all three groups. At the phylum level, the only taxon to meet all three criteria was *Actinobacteria* (Fig. 1c and Supplementary Fig. 1a). We next generated a table of all families represented in this study (Supplementary Table 1). Of these 44 families, only *Erysipelotrichaceae* and *Bifidobacteriaceae* met our criteria, with the relative abundance of both taxa increasing in the AhR ligand-free diet group relative to the conventional diet group and decreasing to similar levels as the conventional diet group upon supplementation of the AhR ligand-free diet with I3C (Fig. 1d and Supplementary Fig. 1b). Because the relative abundance of *Erysipelotrichaceae* in all 3 diet groups was substantially higher than *Bifidobacteriaceae*, and given the previously reported connection between *Erysipelotrichaceae* and AhR^35^, we chose to focus on *Erysipelotrichaceae* rather than *Bifidobacteriaceae* for the remainder of this study.

### *Faecalibaculum rodentium*, an *Erysipelotrichaceae* species, consumes AhR ligands

The results above suggest either that *Erysipelotrichaceae* species favor an environment in which AhR is inactive (and thus the expansion when AhR is not activated by dietary ligands), or that the increase in *Erysipelotrichaceae* relative abundance on the AhR ligand free diet is a compensatory mechanism to generate *Erysipelotrichaceae*-derived AhR ligands when dietary AhR ligands are withheld. In order to perform functional studies to test these opposing hypotheses, we sought to identify an *Erysipelotrichaceae* species from our samples by performing a Basic Local Alignment Search Tool (BLAST) analysis on all of the *Erysipelotrichaceae* 16S rDNA sequences that were among the 100 most abundant OTUs in each of the three groups of mice (Supplementary Tables 2–5). In a combined dataset of all three groups (Supplementary Table 2) and for the conventional diet (Supplementary Table 3) and AhR ligand-free diet (Supplementary Table 4) groups, *Erysipelotrichaceae* was the most abundant OTU, while it was the second most abundant in the AhR ligand-free + I3C diet group (Supplementary Table 5). There were several more occurrences of this family in the top 100 OTU table for all groups, and in all cases BLAST analysis identified their 16S rDNA sequences as most closely matched to *Faecalibaculum rodentium* (Supplementary Tables 2–5). We therefore identified *F*. *rodentium* as a *Erysipelotrichaceae* species in our samples.

**Figure 2:**
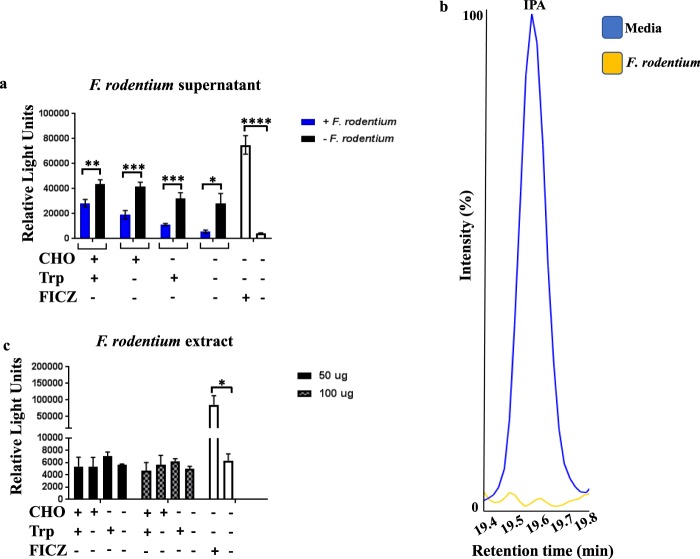
*Faecalibaculum rodentium*, an *Erysipelotrichaceae* species, consumes AhR ligands. (**a**) HCT116 cells engineered to express luciferase upon AhR ligation were treated with bacterial conditioned supernatants from *F*. *rodentium* under the indicated conditions. Cells were also treated with non-conditioned broth to establish baseline levels of any AhR ligands present. As a positive control, some cells were treated with 100 nM of 6-formylindolo[3,2-b]carbazole (FICZ), a known AhR ligand. Dimethyl sulfoxide (DMSO), the FICZ vehicle, was used as a negative control. (**b**) Peak levels of indole-3-propionic acid (IPA) in broth cultures of *F*. *rodentium* cultured in chopped meat broth with carbohydrates and no exogenous Trp. Non-conditioned broth (media) shown for comparison. (**c**) *F*. *rodentium* bead-beaten extracts (total protein amount indicated) failed to induce light production compared with the negative control, indicating a lack of AhR ligands. CHO, carbohydrates. Trp, tryptophan. For a, n = 9 per group. For b, data are representative of 3 experiments. For c, n = 6 per group. Error bars show mean ± SEM. *p < 0.05 **p < 0.01 ***p < 0.001 ****p < 0.0001, Student’s t test.

Members of the gut microbiota, such as *Lactobacillus reuteri*, have been shown to possess the ability to metabolize tryptophan into AhR ligands under carbohydrate-starvation conditions^4^. In order to determine if *F*. *rodentium* consumes or produces AhR ligands, we grew this bacterium under anaerobic conditions in chopped meat broth with or without carbohydrates and supplemented or not with exogenous tryptophan. We also cultured *L*. *reuteri* to serve as a positive control. After 24 hours of exogenous tryptophan treatment, we collected the bacteria-conditioned supernatants and prepared bead-beaten extracts. To measure the amount of AhR ligands present in these samples, we employed HCT116 cells with the luciferase gene under the control of a dioxin response element, allowing these cells to express luciferase upon AhR ligation and translocation to the nucleus. By treating this cell line with bacteria-conditioned supernatants or extracts, we were able to determine the relative levels of AhR ligands in the samples. We also treated our luciferase reporter cell line with broth that had not been conditioned with bacteria but that had been supplemented or not with exogenous tryptophan. Strikingly, the amount of AhR ligands in non-conditioned broth was significantly higher than in the corresponding *F*. *rodentium*-conditioned supernatant in all treatment groups (Fig. 2a).

In order to verify these results, and identify specific AhR ligands consumed by *F*. *rodentium*, we performed a metabolomics analysis on *F*. *rodentium-*conditioned supernatants and non-conditioned broth. We found that the levels of indole-3-propionic acid (IPA), a known AhR ligand^7^, decreased following culturing of *F*. *rodentium* (Fig. 2b). This decrease likely explains the dampened AhR signaling *F*. *rodentium*-conditioned supernatant induced in our luciferase cell reporter assays compared to non-conditioned broth (Fig. 2a).

In contrast to *F*. *rodentium*, while *L*. *reuteri*-conditioned supernatant contained lower levels of AhR ligands than the corresponding non-conditioned broth with carbohydrates and with or without exogenous tryptophan (Supplementary Fig. 2), in carbohydrate-free conditions with exogenous tryptophan added, *L*. *reuteri*-conditioned supernatant contained nearly twice the amount of AhR ligands compared to its corresponding non-conditioned broth, consistent with previous reports^4^. However, this was also true of the carbohydrate-free, no exogenous tryptophan condition, suggesting either that sufficient levels of tryptophan were already present in the broth for *L*. *reuteri* to metabolize into AhR ligands, or that *L*. *reuteri* is capable of metabolizing non-tryptophan sources into AhR ligands during carbohydrate starvation conditions. Indeed, a tryptophan-independent mechanism of AhR ligand production by *L*. *reuteri* was recently described^36^. Interestingly, no AhR ligands were present in the *F*. *rodentium* extracts for any treatment group, as indicated by the lack of light production relative to the negative control (Fig. 2c), suggesting that *F*. *rodentium* metabolizes AhR ligands within 24 hours of uptake, the time period that bacteria were in their final treatment conditions. Taken together, these data support the hypothesis that *F*. *rodentium* favors an environment in which AhR is inactive and is capable of consuming AhR ligands to promote such an environment.

We finally determined if whole fecal microbiota from mice on the three diets differed in their ability to produce or consume AhR ligands. As expected, fecal culture supernatants from mice on the conventional and AhR ligand-free + I3C diets were able to increase AhR signaling relative to non-conditioned culture media (Supplementary Fig. 3). Given our findings discussed above of *F*. *rodentium*-conditioned supernatants failing to stimulate AhR activity to the same level as non-conditioned culture media (Fig. 2a), we hypothesized that supernatants from AhR ligand-free cultures would display a similar deficiency in the ability of activate AhR. Surprisingly, we found microbiota from AhR ligand-free diet mice was able to stimulate AhR signaling to a greater extent than microbiota from either of the other two diets (Supplementary Fig. 3). These results would suggest that removal of AhR ligands from the diet results in a compensatory expansion in members of the microbiota with the ability to generate AhR ligands.

### Fecal microbiota from mice on an AhR ligand-free diet directly reduce secretory IgA levels

**Figure 3:**
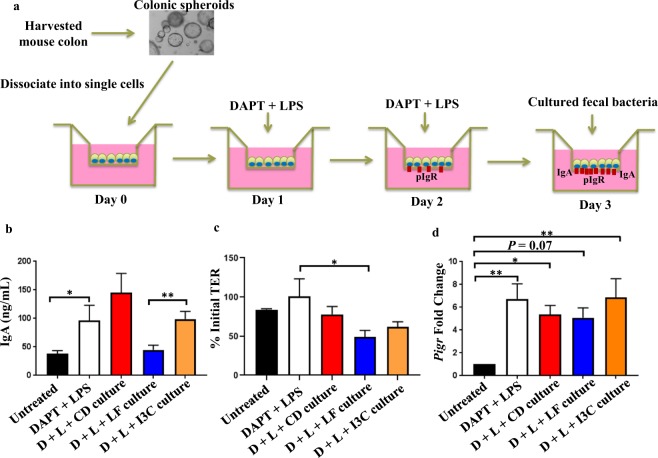
Fecal microbiota from mice on an AhR ligand-free diet directly reduce secretory IgA levels. (**a**) Stem cells were isolated from the epithelial crypts of a mouse colon, cultured as spheroids, dissociated into single cells, and differentiated into epithelial cell monolayers. 100,000 stem cells were seeded into the apical compartment of a Transwell insert on day 0 and treated on days 1 and 2 with 1 µg/mL of lipopolysaccharide (LPS) and 10 µM (N-[N-(3,5-difluorophenacetyl-L-alanyl)]-S-phenylglycine t-butyl ester (DAPT) to induce expression of pIgR and differentiate the cells, respectively. As a negative control, some cells were not treated with LPS and DAPT (untreated). On day 3, cells were removed from their treatments and placed in fresh medium. IgA was added to the basal compartment of the Transwell at a concentration of 5 µg/mL. At the same time, bacterial fecal cultures from mice on the conventional diet (CD culture), AhR ligand-free diet (LF culture), or AhR ligand-free diet supplemented with I3C (I3C culture) were added to the apical compartment at a 1/10 dilution. (**b**) Medium in the apical compartment was collected 6 hours later and IgA levels assessed by ELISA. (**c**) On day 3, transepithelial electrical resistance (TER) of monolayers was measured immediately before adding fecal bacterial cultures. A second TER measurement was obtained 6 hours later. The data show this second TER measurement expressed as a percentage of the initial TER measurement. (**d**) Immediately after collection of apical supernatants and final TER measurements, cells were lysed in Trizol reagent, and *Pigr* expression was quantified by qPCR. Fold change in *Pigr* expression is relative to the untreated group. D, DAPT. L, LPS. For b, n = 5–8 per group. For c, n = 3–5 per group. For d, n = 3–6 per group. Error bars show mean ± SEM. *p < 0.05 **p < 0.01, one-way ANOVA with Tukey’s multiple comparisons test.

Having shown an association between altered microbiota composition and reduced fecal IgA levels in mice on the AhR ligand-free diet, we next sought to determine if a direct cause-and-effect relationship existed. To do so, we added fecal cultures obtained from mice on either the conventional, AhR ligand-free, or AhR ligand-free + I3C diets to the apical surface of primary colonic epithelial cell monolayers differentiated from epithelial stem cell spheroids in order to model IgA transcytosis^34^. During IgA transcytosis, polymeric IgA that is secreted by plasma cells in the lamina propria binds to the polymeric Ig receptor (pIgR) on the basolateral side of the epithelium^37–39^. The pIgA-pIgR complex is then endocytosed and transported across the cell to the apical surface^40–42^. Cellular enzymes cleave the receptor, releasing secretory IgA (SIgA) into the lumen. However, SIgA is still bound to the extracellular portion of pIgR, known as secretory component, which makes SIgA less susceptible to cleavage by bacterial proteases^43^. In our *in vitro* model, primary epithelial stem cells from cultured spheroids were seeded in a Transwell insert and allowed to form into epithelial cell monolayers. After treatment with (*N*-[*N*-(3,5-difluorophenacetyl-L-alanyl)]-*S*-phenylglycine *t*-butyl ester (DAPT) and lipopolysaccharide (LPS) to promote cell differentiation and pIgR expression, respectively, IgA was added to the basal compartment of the Transwell at the same time as the bacterial cultures were added to the apical compartment (Fig. 3a). Six hours later, apical supernatants were collected and analyzed for IgA concentration.

As expected, higher concentrations of IgA were observed in the apical supernatants of monolayers that were treated with DAPT and LPS compared with monolayers that were left untreated (Fig. 3b). Addition of bacterial cultures from conventional diet mice did not significantly alter the IgA concentration compared with DAPT and LPS treatment alone, whereas addition of bacterial cultures from AhR ligand-free mice led to an apical IgA concentration comparable to that observed from untreated monolayers (Fig. 3b). Importantly, addition of AhR ligand-free + I3C cultures resulted in apical IgA concentrations that were intermediate between those seen upon addition of conventional diet and AhR ligand-free cultures (Fig. 3b). IgA transcytosis requires an intact monolayer. If the physical integrity of the monolayer is compromised, IgA can passively diffuse across the membrane of the Transwell insert rather than actively being transcytosed through the epithelial cells. Therefore, in order to rule out the possibility that our results could be explained by differential effects of the fecal cultures on monolayer integrity, we measured transepithelial electrical resistance (TER) immediately before the addition of fecal cultures, followed by a second TER measurement six hours later. We found no significant differences in TER for any of the fecal cultures (Fig. 3c). Furthermore, it is unlikely that the decrease in apical IgA concentration observed upon addition of AhR ligand-free cultures can be explained by a deficiency in IgA transcytosis, because expression of *Pigr* among all the groups treated with DAPT and LPS was similar (Fig. 3d). We also observed a trend of increased fecal IgA concentration from mice on the AhR ligand-free + I3C diet compared to mice on the AhR ligand-free diet, although this increase did not reach statistical significance (Supplementary Fig. 4).

### F. rodentium secretes factors that modulate SIgA levels

Finally, we sought to determine if a causative relationship exists between our observation of increased fecal *Erysipelotrichaceae* relative abundance and decreased fecal IgA in mice on an AhR ligand-free diet compared with mice on a conventional diet. To do so, we added *F*. *rodentium* bead-beaten extracts or conditioned supernatant to the apical compartment in our *in vitro* IgA transcytosis model. Six hours later, apical supernatants were collected.

**Figure 4:**
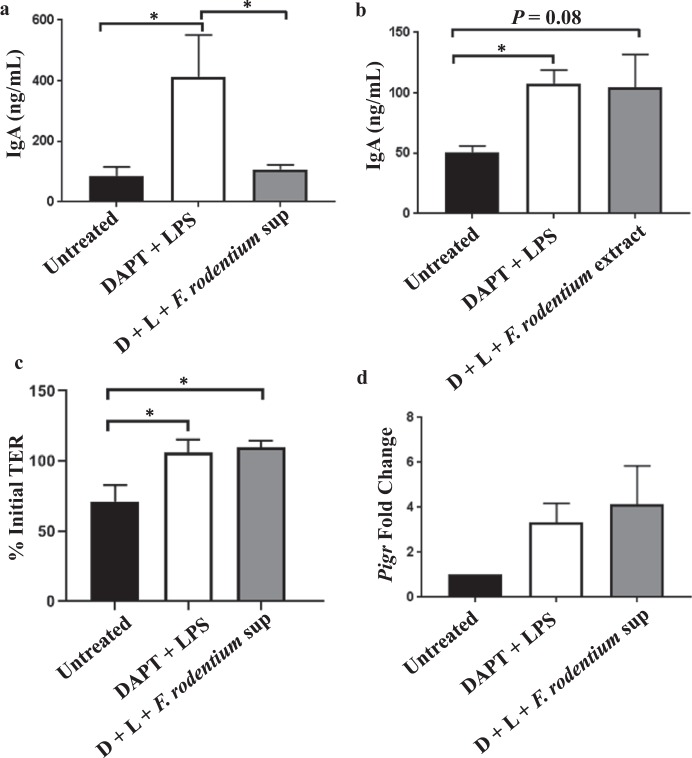
*F*. *rodentium* secretes factors that modulate SIgA levels. Stem cell spheroids were cultured and differentiated into epithelial cell monolayers as described in Fig. 3a. On day 3, *F*. *rodentium*-conditioned supernatant (**a**) or bead-beaten extracts (**b**) were added to the apical compartment of a Transwell insert at a 1/10 dilution, and 5 µg/mL IgA was added to the basal compartment. Six hours later, apical supernatants were collected and IgA levels assessed by ELISA. (**c**) An initial and final TER measurement was obtained as described in Fig. 3c. (**d**) Immediately after collection of apical supernatants and final TER measurements, cells from (a) were lysed in Trizol reagent and *Pigr* expression was quantified by qPCR. Fold change in *Pigr* expression is relative to the untreated group. D, DAPT. L, LPS. sup, supernatant. Error bars show mean ± SEM. For a, n = 8–9 per group. For b, n = 3 per group. For c, n = 3–5 per group. For d, n = 3–4 per group. *p < 0.05, one-way ANOVA with Tukey’s multiple comparisons test.

As expected, we observed a higher concentration of IgA in the apical supernatants of monolayers treated with DAPT and LPS compared with monolayers that were left untreated (Fig. 4a,b). Addition of *F*. *rodentium*-conditioned supernatant to monolayers treated with DAPT and LPS led to a significant decrease in apical IgA concentration to levels comparable to the apical concentration seen in untreated monolayers. (Fig. 4a). In contrast to *F*. *rodentium*-conditioned supernatant, addition of *F*. *rodentium* bead-beaten extracts had no effect on the levels of apical IgA (Fig. 4b), suggesting factors secreted by *F*. *rodentium*, rather than cellular contents, are responsible for the observed decrease in apical IgA. Importantly, the ability of *F*. *rodentium* supernatant to lower apical IgA levels cannot be attributed to differential effects on TER (Fig. 4c) or *Pigr* expression (Fig. 4d) relative to DAPT + LPS treatment alone.

One possible interpretation of the collective results of Figs 3 and 4 is that microbiota from mice on the AhR ligand-free diet, and *F*. *rodentium* specifically, have the ability to degrade IgA. We attempted to perform immunoblotting on the apical supernatants to possibly visualize fragmented IgA, but the high amount of protein in chopped meat broth prevented us from doing so.

## Discussion

Here we report that a diet devoid of AhR ligands leads to a fundamentally reshaped fecal microbiota, including an increase in the relative abundance of the family *Erysipelotrichaceae*. Even though addition of a single AhR ligand, I3C, to the AhR ligand-free diet was insufficient to completely restore the global microbiota composition to that of mice on a conventional diet, it was nevertheless sufficient to lower *Erysipelotrichaceae* relative abundance to a level similar to that observed in mice on a conventional diet.

There are conflicting reports regarding the potential beneficial or harmful effects of this family in inflammatory gastrointestinal disease. Ileitis-associated increases in *Erysipelotrichaceae* abundance have been reported in mice infected with *Toxoplasma gondii* or *Giardia muris*^44^, and this family is also expanded in a murine model of tumor necrosis factor (TNF)-driven Crohn’s disease (CD)-like ileitis^45^. In contrast, lower *Erysipelotrichaceae* abundance in humans has been associated with new-onset CD^46^ and recurring CD^47^. Collectively, these discrepancies might partially be explained by inherent differences in the gut microbiota between mice and humans^48^ and differential effects exerted by individual *Erysipelotrichaceae* species. Compared to gastrointestinal inflammation, there is more agreement concerning the relationship between *Erysipelotrichaceae* and metabolic disorders. Specifically, *Erysipelotrichaceae* abundance is elevated in mouse models of diet-induced obesity^49–51^ and in obese individuals^52^. On the other hand, low abundance of *Erysipelotrichaceae* has been associated with an increased chance of developing type 1 diabetes in the non-obese diabetic mouse model^53^.

Interestingly, it has been reported that mice fed a diet supplemented with broccoli, a rich source of AhR ligands, harbor a reduced abundance of *Erysipelotrichaceae*^35^. Our results provide evidence for the inverse as well: that a diet depleted of AhR ligands leads to an expansion of this bacterial family.

Given the beneficial and harmful properties associated with *Erysipelotrichaceae*, we sought to clarify in our own model whether or not the observed increase in *Erysipelotrichaceae* in mice on the AhR ligand-free diet represented a potentially beneficial or harmful event. In order to so, we first identified *F*. *rodentium* as the species most closely related to the *Erysipelotrichaceae* 16S rDNA sequences from mice in all three diets. We found that *F*. *rodentium* depletes its growth medium of AhR ligands. Furthermore, addition of *F*. *rodentium*-conditioned medium to the apical epithelial surface in an *in vitro* model of IgA transcytosis results in a decrease in apical IgA concentration. Based on these findings, we speculate that an increase in *F*. *rodentium* abundance in the context of dietary AhR ligand deficiency would represent an adverse event to the health of the host. However, whole microbiota from mice on the AhR ligand-free diet were able to recapitulate the IgA-reducing, but not the AhR ligand consuming, effects of *F*. *rodentium*. Given the greater susceptibility of mice with low fecal IgA levels to chemically-induced colitis^30,54^, future work will determine if the AhR ligand-free diet makes mice more susceptible to DSS colitis and if the microbiota in general and *F*. *rodentium* in particular directly contribute to the higher susceptibility.

As an essential amino acid, tryptophan cannot be synthesized by the host but must come from the diet. In the gastrointestinal tract, tryptophan can be directly metabolized into AhR ligands by the gut microbiota^4^. These microbial-derived ligands consist largely of indole derivatives^2,7^. Fecal samples from germ-free mice are deficient for AhR ligands^8^, underscoring the essential contribution made by the microbiota in regulating AhR activity. However, despite the well-established connection between commensal bacteria AhR ligand production and AhR physiological function^55^, the effects of disrupted AhR signaling on the composition and function of the gut microbiota has been understudied. Many studies involving suppression of AhR activity involve knockout of *Ahr*. However, we elected to investigate the effect of deficient AhR activity on the microbiota by instead withholding dietary AhR ligands, which represents a more physiologically relevant situation in terms of translational research. Our results utilizing cultured fecal bacteria from mice on the conventional, AhR ligand-free, and AhR ligand-free + I3C diets in our *in vitro* model of IgA transcytosis provide evidence that the reshaped fecal microbiota upon withholding of dietary AhR ligands is capable of directly decreasing IgA levels in the intestinal lumen. We propose that the *Erysipelotrichaceae* species *F*. *rodentium* is a contributor to this effect, because of our results incorporating *F*. *rodentium*-conditioned supernatant in our *in vitro* IgA transcytosis model and the decrease in *Erysipelotrichaceae* following supplementation of the AhR ligand-free diet with I3C.

Given our previous finding of reduced B cell numbers in intestinal Peyer’s patches from mice on the AhR ligand-free diet^14^, we cannot rule out the possibility that a deficiency in B cell gut recruitment and/or function contributes to lower fecal IgA levels in mice with no dietary sources of AhR ligands. Potential mechanisms linking AhR activation to B cell secretion of IgA is a source of ongoing work in our laboratory.

Dietary sources of AhR ligands fall into two main categories: flavonoids and other phytochemicals and tryptophan derivatives. Flavonoids are polyphenolic compounds found in a variety of fruits, vegetables, and plant-derived beverages such as tea and wine^5^. On the other hand, indole molecules, including I3C, are tryptophan derivatives. I3C is a metabolite of glucosinolate glucobrassicin, a major component of cruciferous vegetables^56^. Importantly, I3C and its derivatives possess anti-inflammatory properties in the setting of experimental systemic lupus erythematosus^57^ and chemically-induced colitis^58^. Although in the present study we supplemented the AhR ligand-free diet with only I3C, it would be of interest to investigate the effects other AhR dietary ligands exert on the microbiota.

We readily acknowledge our study has limitations. We were unable to show a statistically significant effect of I3C, when added to the AhR ligand-free diet, on fecal IgA levels. A relatively low sample size and high variability likely contributed to this failure, but the tendency of I3C supplementation to raise fecal IgA levels is clearly present. In addition, most of the *Erysipelotrichaceae* DNA sequences were not a 100% match to *F*. *rodentium*, even though *F*. *rodentium* was the closest possible species-level match according to our BLAST analysis. Nevertheless, it is possible that no actual AhR ligand-consuming taxa are present in the microbiota of mice on the AhR ligand-free diet, even if *F*. *rodentium* itself does have this characteristic. Such a scenario would explain our discordant results from the luciferase cell reporter assay utilizing culture supernatants of *F*. *rodentium* alone versus fecal samples from mice on the AhR ligand-free diet.

In conclusion, our results point to dietary AhR ligands as key factors in shaping the composition of the gut microbiota. A diet deficient in AhR ligands leads to an altered microbial community capable of decreasing IgA levels, an event that potentially increases susceptibility to colitis. *F*. *rodentium* was identified as likely contributing to IgA level modulation. Our data indicate that a diet rich in I3C might promote intestinal health by maintaining proper gut IgA levels through regulation of *F*. *rodentium* abundance. To our knowledge, this is the first time a specific bacterial commensal species has been implicated in modulating levels of AhR ligands and fecal IgA.

## Supplementary information

Supplementary Material

## Data Availability

All microbiota DNA sequences generated in this study have been deposited in the Sequence Read Archive under accession number PRJNA510099.
