# Supplementary material for: Depletion of dietary aryl hydrocarbon receptor ligands alters microbiota composition and function
Source: Sci Rep. 2019 Oct 11;9:14724. doi: 10.1038/s41598-019-51194-w (PMC6789125; doi:10.1038/s41598-019-51194-w)
Supplement: Supplementary file 1 — Supplementary Material [file 41598_2019_51194_MOESM1_ESM.pdf]

Supplementary Material

Depletion of dietary aryl hydrocarbon receptor ligands alters microbiota composition and function

Kyle M. Brawner*[1], Venkata A. Yeramilli[1], Lennard W. Duck[2], William Van Der Pol[3], Lesley E. Smythies[2], Casey D. Morrow[4], Charles O. Elson[2], & Colin A. Martin[1]

[1]Department of Surgery, [2]Department of Medicine, [3]Center for Clinical and Translational Science, and [4]Department of Cell, Developmental, and Integrative Biology, University of Alabama at Birmingham, Birmingham, Alabama, United States.

*Corresponding Author:
Kyle M. Brawner, Ph.D.
Postdoctoral Fellow
Department of Surgery
University of Alabama at Birmingham
1600 7th Ave. S., Lowder Building Suite 300
Birmingham, AL 35233
Tel: 205-934-3152
kbrawner@uab.edu

Supplementary Figure 1

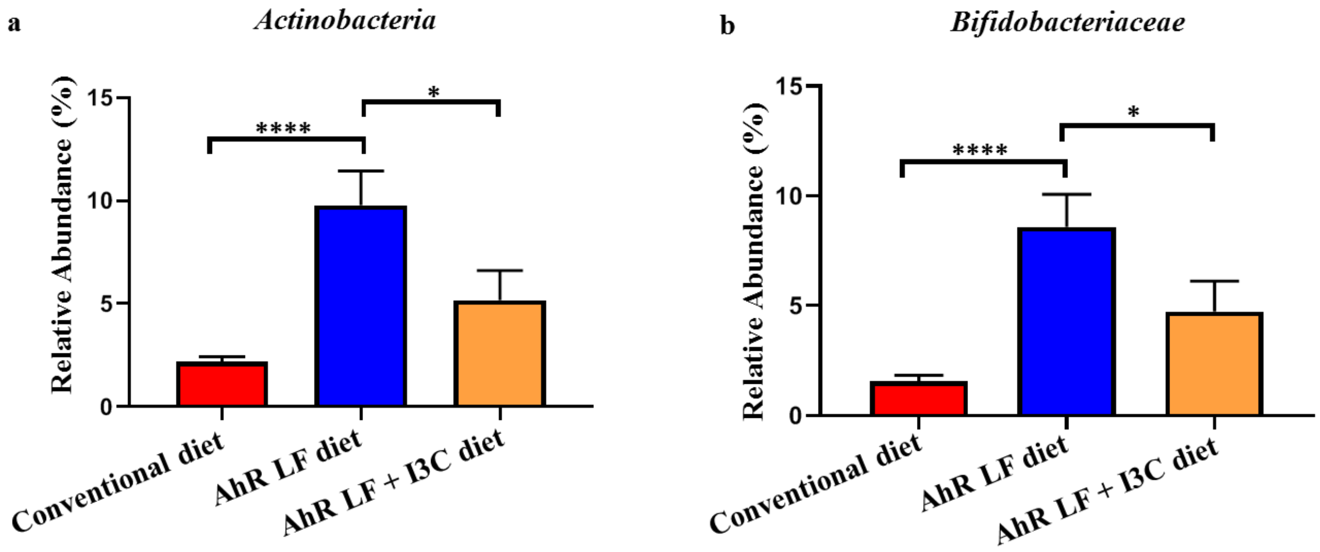

Relative abundance of (**a**) *Actinobacteria* and (**b**) *Bifidobacteriaceae* from mice on the conventional diet (n=12), mice on the AhR ligand-free (LF) diet (n=7), and mice on the AhR LF diet supplemented with I3C (AhR LF + I3C diet) (n=6). Error bars show mean $\pm$ SEM. *p < 0.05 ****p < 0.0001, one-way ANOVA with Tukey's multiple comparisons test.

Supplementary Figure 2

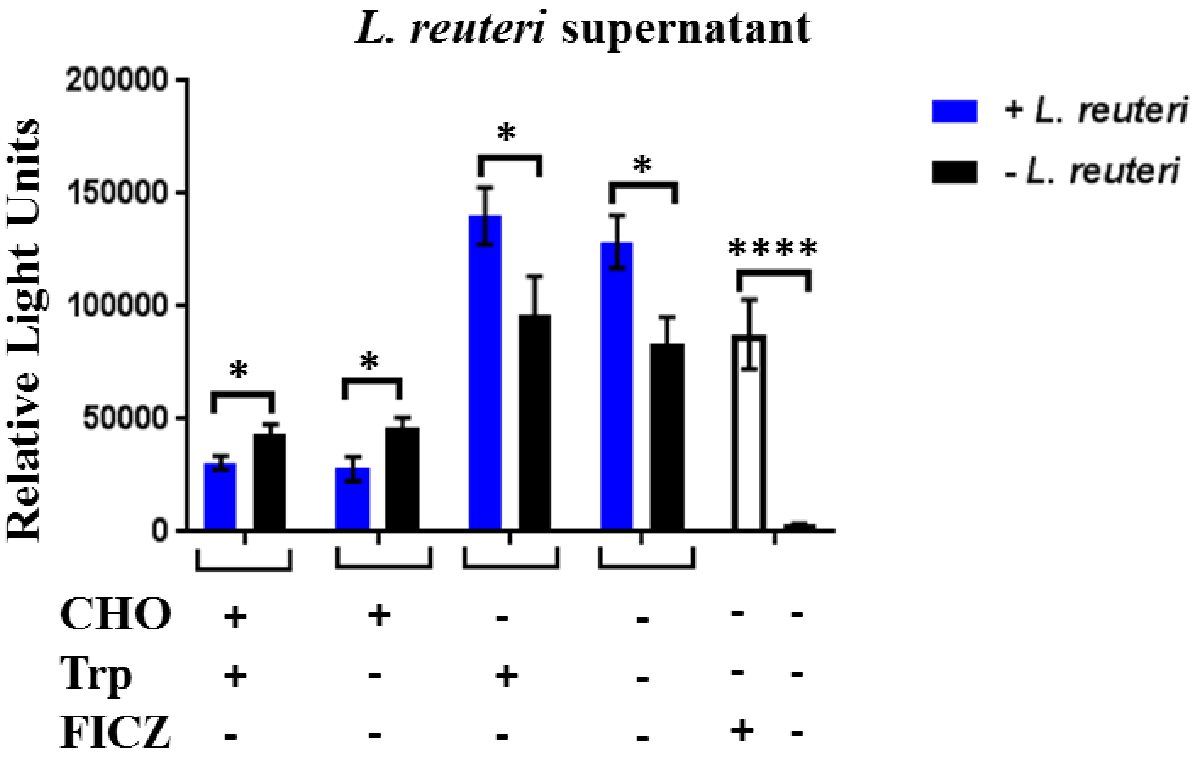

HCT116 cells engineered to express luciferase upon AhR ligation were treated with bacterial conditioned supernatants from *Lactobacillus reuteri* cultured under the indicated conditions. Cells were also treated with non-conditioned broth to establish baseline levels of any AhR ligands present. As a positive control, some cells were treated with 100nM of 6-formylindolo[3,2-b]carbazole (FICZ), a known AhR ligand. Dimethyl sulfoxide (DMSO), the FICZ vehicle, was used as a negative control. n=9 per group. Error bars show mean ± SEM. *p < 0.05 ****p < 0.0001, Student's t test.

Supplementary Figure 3

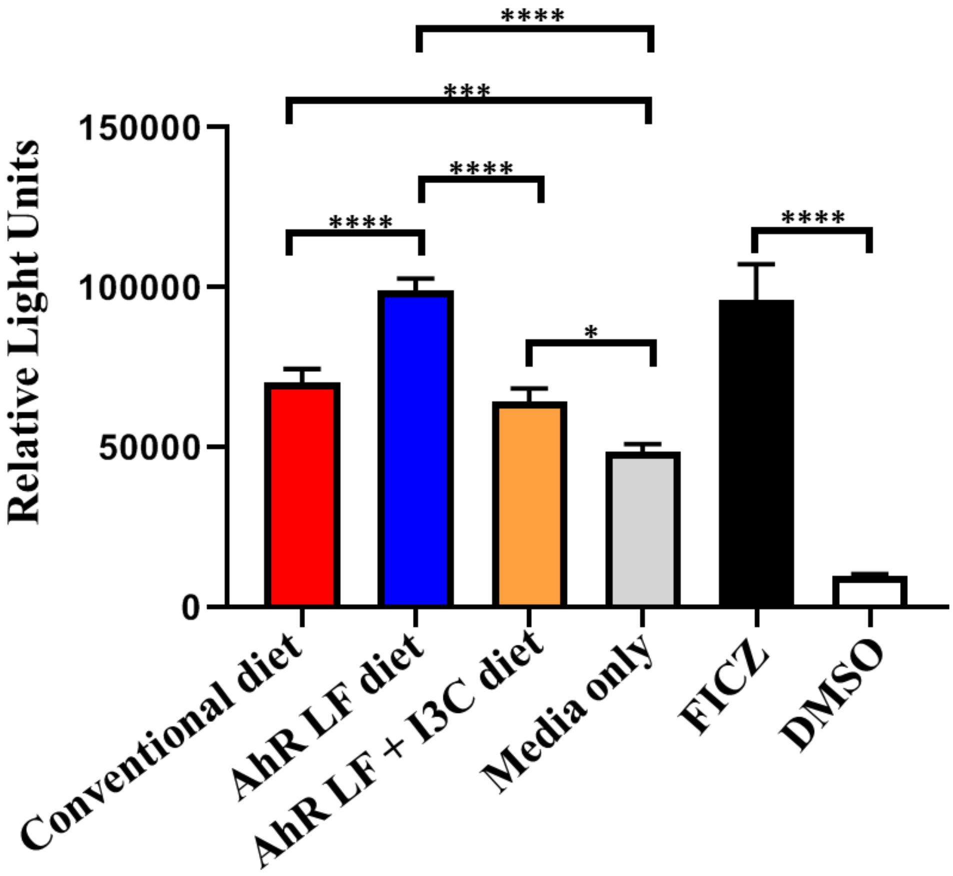

Culture supernatants of fecal samples obtained from mice on the indicated diet were cultured with HCT116 cells that express luciferase when AhR is activated. HCT116 cells were also treated with sterile chopped meat broth (media only) to evaluate how initial levels of AhR ligands in the broth are altered following fecal culture. As a positive control, some cells were treated with 100nM of 6-formylindolo[3,2-b]carbazole (FICZ), a known AhR ligand. Dimethyl sulfoxide (DMSO), the FICZ vehicle, was used as a negative control. n=7 or 8 per group. Error bars show mean $\pm$ SEM. *$p<0.05$ ***$p<0.001$ ****$p<0.0001$, one-way ANOVA with Tukey's multiple comparisons test.

Supplementary Figure 4

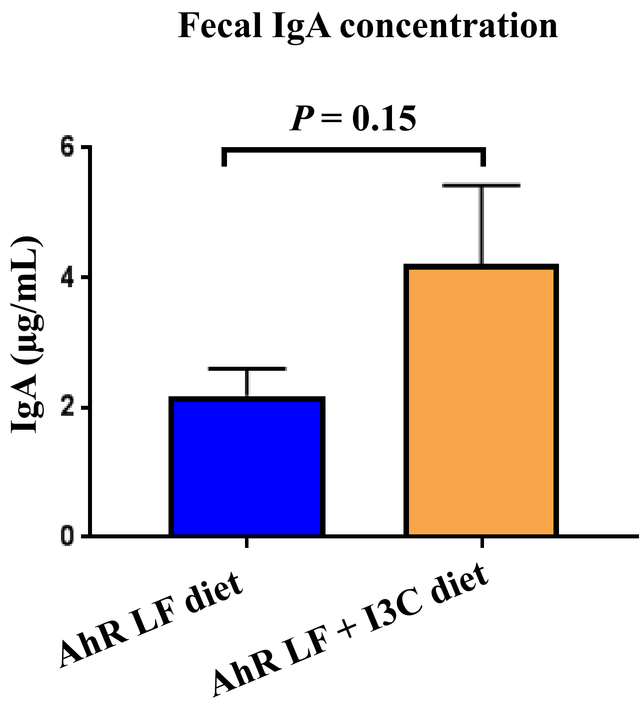

Fecal samples from mice on the AhR ligand-free (LF) diet and AhR LF diet supplemented with I3C (AhR LF + I3C diet) were collected and IgA levels were determined by ELISA. n=5 for each group. Error bars show mean $\pm$ SEM. *P* value was obtained from a two-tailed, unpaired Student's t test.

Supplementary Table 1

Relative abundance of bacterial families among each diet and in all mice combined

	CD average	LF average	I3C average	Overall average
k__Bacteria;p__Firmicutes;c__Erysipelotrichi;o__Erysipelotrichales;f__Erysipelotrichaceae	0.176450108	0.464700659	0.247591048	0.274234088
k__Bacteria;p__Bacteroidetes;c__Bacteroidia;o__Bacteroidales;f__S24-7	0.199449395	0.123568204	0.128024563	0.161060702
k__Bacteria;p__Verrucomicrobia;c__Verrucomicrobiae;o__Verrucomicrobiales;f__Verrucomicrobiaceae	0.057919947	0.130534882	0.319514684	0.141034866
k__Bacteria;p__Firmicutes;c__Clostridia;o__Clostridiales;f__Lachnospiraceae	0.128443678	0.025282454	0.037914195	0.077831459
k__Bacteria;p__Firmicutes;c__Bacilli;o__Lactobacillales;f__Lactobacillaceae	0.128367083	0.029214056	0.020014263	0.074599559
k__Bacteria;p__Firmicutes;c__Clostridia;o__Clostridiales;Other	0.038259267	0.023601923	0.081191576	0.044458965
k__Bacteria;p__Actinobacteria;c__Actinobacteria;o__Bifidobacteriales;f__Bifidobacteriaceae	0.015458435	0.085793387	0.047286109	0.042790863
k__Bacteria;p__Proteobacteria;c__Deltaproteobacteria;o__Desulfovibrionales;f__Desulfovibrionaceae	0.022562838	0.034865496	0.026801647	0.027024897
k__Bacteria;p__Firmicutes;c__Clostridia;o__Clostridiales;f__	0.04845347	0.006001074	0.007001933	0.02661843
k__Bacteria;p__Firmicutes;c__Clostridia;o__Clostridiales;f__Ruminococcaceae	0.02074361	0.023537134	0.031336934	0.024068194
k__Bacteria;p__Firmicutes;c__Bacilli;o__Turicibacterales;f__Turicibacteraceae	0.048257014	0.001901098	7.73806E-05	0.023714245
k__Bacteria;p__Firmicutes;c__Clostridia;o__Clostridiales;f__Clostridiaceae	0.036399596	6.28506E-05	0.000146797	0.017524636
k__Bacteria;p__Bacteroidetes;c__Bacteroidia;o__Bacteroidales;f__Prevotellaceae	0.022596605	0.000283726	0.001100646	0.011189969
k__Bacteria;p__Bacteroidetes;c__Bacteroidia;o__Bacteroidales;f__Rikenellaceae	0.0140724	0.002557747	0.011112188	0.010137846
k__Bacteria;p__Firmicutes;Other;Other;Other	0.016681076	0.003885122	0.001619814	0.009483506
k__Bacteria;p__Actinobacteria;c__Coriobacteriia;o__Coriobacteriales;f__Coriobacteriaceae	0.006404491	0.012066181	0.004457819	0.007522563
k__Bacteria;p__Bacteroidetes;c__Bacteroidia;o__Bacteroidales;f__Bacteroidaceae	0.007953848	0.006178197	0.006041413	0.006997682
k__Bacteria;p__Firmicutes;c__Bacilli;o__Lactobacillales;f__Streptococcaceae	0.000717262	0.003815807	0.009694182	0.003739316
k__Bacteria;p__Proteobacteria;c__Alphaproteobacteria;o__Rickettsiales;f__	0.001562048	0.003075664	0.003880468	0.002542281
k__Bacteria;p__Bacteroidetes;c__Bacteroidia;o__Bacteroidales;f__Porphyromonadaceae	0.00017968	0.00700729	0.000463006	0.002159409
k__Bacteria;Other;Other;Other;Other	0.000308934	0.004205754	0.001449977	0.001673894
k__Bacteria;p__Firmicutes;c__Clostridia;Other;Other	0.000196381	0.002377021	0.00335733	0.001565588
k__Bacteria;p__Bacteroidetes;c__Bacteroidia;o__Bacteroidales;f__[Odoribacteraceae]	0.00010789	0.001431372	0.004161956	0.001451441
k__Bacteria;p__Proteobacteria;c__Gammaproteobacteria;o__Pseudomonadales;f__Moraxellaceae	0.001897397	0.000737582	2.16084E-05	0.00112246
k__Bacteria;p__Firmicutes;c__Clostridia;o__Clostridiales;f__[Mogibacteriaceae]	0.001446969	0.000246997	0.00040885	0.000861828
k__Bacteria;p__Proteobacteria;c__Alphaproteobacteria;o__RF32;f__	0.00075304	0.00034592	0.000973969	0.000692069
k__Bacteria;p__Cyanobacteria;c__4C0d-2;o__YS2;f__	0	6.88777E-05	0.002253691	0.000560172
k__Bacteria;p__Proteobacteria;c__Betaproteobacteria;o__Burkholderiales;f__Alcaligenaceae	0.000786575	0.000104741	0.000135577	0.000439422
k__Bacteria;p__Firmicutes;c__Bacilli;Other;Other	0.00080704	6.58215E-05	2.71165E-05	0.000412317
k__Bacteria;p__Firmicutes;c__Clostridia;o__Clostridiales;f__Dehalobacteriaceae	0.00034323	0.000182849	0.000758441	0.000397974
k__Bacteria;p__Bacteroidetes;c__Bacteroidia;o__Bacteroidales;f__	2.83688E-06	0.000874319	1.05299E-05	0.000248698
k__Bacteria;p__Proteobacteria;c__Gammaproteobacteria;o__Enterobacteriales;f__Enterobacteriaceae	0.000331185	5.859E-05	0.000235146	0.000231809
k__Bacteria;p__Firmicutes;c__Bacilli;o__Lactobacillales;Other	0.000326591	0.000166859	4.88147E-05	0.0002152
k__Bacteria;p__Proteobacteria;c__Betaproteobacteria;o__Burkholderiales;f__Comamonadaceae	0.000297337	0.000147422	0.000103714	0.000208891
k__Bacteria;p__Tenericutes;c__Mollicutes;o__RF39;f__	0.000172895	0.000349109	1.05299E-05	0.000183267
k__Bacteria;p__Firmicutes;c__Clostridia;o__Clostridiales;f__Veillonellaceae	0.000232961	5.15748E-05	0.00014622	0.000161355
k__Bacteria;p__Proteobacteria;c__Gammaproteobacteria;o__Pseudomonadales;f__Pseudomonadaceae	0.000194941	9.67035E-05	9.19042E-05	0.000142706
k__Bacteria;p__Firmicutes;c__Bacilli;o__Bacillales;f__Staphylococcaceae	0.000138951	0.000126464	6.55179E-05	0.000117831
k__Bacteria;p__Firmicutes;c__Clostridia;o__Clostridiales;f__Eubacteriaceae	0.000230757	9.06483E-06	1.64793E-05	0.000117257
k__Bacteria;p__Tenericutes;c__Mollicutes;o__Anaeroplasmatales;f__Anaeroplasmataceae	0.00023811	0	1.08243E-05	0.000116891
k__Bacteria;p__Bacteroidetes;c__Bacteroidia;o__Bacteroidales;f__[Barnesiellaceae]	0.000211246	2.34583E-05	1.06807E-05	0.00011053
k__Bacteria;p__Firmicutes;c__Clostridia;o__Clostridiales;f__Christensenellaceae	2.8577E-06	0.00033745	0	9.58576E-05
k__Bacteria;p__Firmicutes;c__Clostridia;o__Clostridiales;f__Peptococcaceae	4.00236E-05	0	0.000262141	8.21252E-05
k__Bacteria;p__Firmicutes;c__Clostridia;o__Clostridiales;f__Peptostreptococcaceae	0	5.91006E-05	0.000168317	5.69443E-05

Families are ordered from the highest to lowest overall relative abundance average (the average relative abundance of all mice in the study). Also shown is the relative abundance for each diet. CD, conventional diet (n=12). LF, AhR ligand-free diet (n=7). I3C, AhR ligand-free diet supplemented with I3C (n=6).

Supplementary Table 2

Characterization of the top 100 most abundant *Erysipelotrichaceae* operational taxonomic units (all 3 groups combined)

OTU abundance rank	Consensus lineage	Average relative abundance (all samples, %)	Top BLAST result (percent match)
1	k__Bacteria; p__Firmicutes; c__Erysipelotrichi; o__Erysipelotrichales; f__Erysipelotrichaceae	20.7	Faecalibaculum rodentium strain ALO17 (88%)
6	k__Bacteria; p__Firmicutes; c__Erysipelotrichi; o__Erysipelotrichales; f__Erysipelotrichaceae	3.7	Faecalibaculum rodentium strain ALO17 (93%)
11	k__Bacteria; p__Firmicutes; c__Erysipelotrichi; o__Erysipelotrichales; f__Erysipelotrichaceae	1.67	Faecalibaculum rodentium strain ALO17 (100%)
64	k__Bacteria; p__Firmicutes; c__Erysipelotrichi; o__Erysipelotrichales; f__Erysipelotrichaceae	0.138	Faecalibaculum rodentium strain ALO17 (90%)
75	k__Bacteria; p__Firmicutes; c__Erysipelotrichi; o__Erysipelotrichales; f__Erysipelotrichaceae	0.109	Faecalibaculum rodentium strain ALO17 (92%)
80	k__Bacteria; p__Firmicutes; c__Erysipelotrichi; o__Erysipelotrichales; f__Erysipelotrichaceae	0.101	Faecalibaculum rodentium strain ALO17 (97%)

Supplementary Table 3

Characterization of the top 100 most abundant *Erysipelotrichaceae* operational taxonomic units within the conventional diet group

OTU abundance rank	Consensus lineage	Average relative abundance (all samples, %)	Top BLAST result (% match)
1	k__Bacteria; p__Firmicutes; c__Erysipelotrichi; o__Erysipelotrichales; f__Erysipelotrichaceae	12.6	Faecalibaculum rodentium strain ALO17 (88)
8	k__Bacteria; p__Firmicutes; c__Erysipelotrichi; o__Erysipelotrichales; f__Erysipelotrichaceae	3.4	Faecalibaculum rodentium strain ALO17 (93)
31	k__Bacteria; p__Firmicutes; c__Erysipelotrichi; o__Erysipelotrichales; f__Erysipelotrichaceae	0.43	Faecalibaculum rodentium strain ALO17 (100)
56	k__Bacteria; p__Firmicutes; c__Erysipelotrichi; o__Erysipelotrichales; f__Erysipelotrichaceae	0.18	Faecalibaculum rodentium strain ALO17 (92)
78	k__Bacteria; p__Firmicutes; c__Erysipelotrichi; o__Erysipelotrichales; f__Erysipelotrichaceae	0.074	Faecalibaculum rodentium strain ALO17 (90)
92	k__Bacteria; p__Firmicutes; c__Erysipelotrichi; o__Erysipelotrichales; f__Erysipelotrichaceae	0.00719	Faecalibaculum rodentium strain ALO17 (97)

Supplementary Table 4

Characterization of the top 100 most abundant *Erysipelotrichaceae* operational taxonomic units within the AhR ligand-free diet group

OTU abundance rank	Consensus lineage	Average relative abundance (all samples, %)	Top BLAST result (% match)
1	k__Bacteria; p__Firmicutes; c__Erysipelotrichi; o__Erysipelotrichales; f__Erysipelotrichaceae	37.6	Faecalibaculum rodentium strain ALO17 (88)
5	k__Bacteria; p__Firmicutes; c__Erysipelotrichi; o__Erysipelotrichales; f__Erysipelotrichaceae	3.6	Faecalibaculum rodentium strain ALO17 (93)
6	k__Bacteria; p__Firmicutes; c__Erysipelotrichi; o__Erysipelotrichales; f__Erysipelotrichaceae	3.4	Faecalibaculum rodentium strain ALO17 (100)
21	k__Bacteria; p__Firmicutes; c__Erysipelotrichi; o__Erysipelotrichales; f__Erysipelotrichaceae	0.29	Faecalibaculum rodentium strain ALO17 (90)
23	k__Bacteria; p__Firmicutes; c__Erysipelotrichi; o__Erysipelotrichales; f__Erysipelotrichaceae	0.27	Faecalibaculum rodentium strain ALO17 (97)
75	k__Bacteria; p__Firmicutes; c__Erysipelotrichi; o__Erysipelotrichales; f__Erysipelotrichaceae	0.014	Faecalibaculum rodentium strain ALO17 (92)

Supplementary Table 5

Characterization of the top 100 most abundant *Erysipelotrichaceae* operational taxonomic units within the AhR ligand-free + I3C diet group

OTU abundance rank	Consensus lineage	Average relative abundance (all samples, %)	Top BLAST result (% match)
2	k__Bacteria; p__Firmicutes; c__Erysipelotrichi; o__Erysipelotrichales; f__Erysipelotrichaceae	16.9	Faecalibaculum rodentium strain ALO17 (88)
5	k__Bacteria; p__Firmicutes; c__Erysipelotrichi; o__Erysipelotrichales; f__Erysipelotrichaceae	4.4	Faecalibaculum rodentium strain ALO17 (93)
8	k__Bacteria; p__Firmicutes; c__Erysipelotrichi; o__Erysipelotrichales; f__Erysipelotrichaceae	2.1	Faecalibaculum rodentium strain ALO17 (100)
56	k__Bacteria; p__Firmicutes; c__Erysipelotrichi; o__Erysipelotrichales; f__Erysipelotrichaceae	0.0914	Faecalibaculum rodentium strain ALO17 (97)
59	k__Bacteria; p__Firmicutes; c__Erysipelotrichi; o__Erysipelotrichales; f__Erysipelotrichaceae	0.0802	Faecalibaculum rodentium strain ALO17 (90)
60	k__Bacteria; p__Firmicutes; c__Erysipelotrichi; o__Erysipelotrichales; f__Erysipelotrichaceae	0.0802	Faecalibaculum rodentium strain ALO17 (92)
